# Splitting of a Tooth by Decomposition of the Pulp

**Published:** 1872-01

**Authors:** J. H. M'Quillen


### ARTICLE VIII.

### *Splitting of a Tooth by Decomposition of the Pulp.*

BY J. H. M'QUILLEN, M.D., D.D.S.

C. G., Esq., called upon me in the first part of October, 1871, complaining of severe pain in the right superior first bicuspid; the pain had continued for several days, and had been a constant source of annoyance. On examination, the tooth was found perfectly sound, with not the slightest evidence of any defect of structure, or decay; the gum around it, however, was somewhat inflamed and swollen, and on striking the tooth with an instrument, or on the occlusion of the jaws, the pain was increased in intensity.

After a careful consideration of the case, I stated to the patient that I had reason to believe that his difficulty had its origin in the calcification of the bulbous portion of the pulp of the tooth, exhibiting to him at the same time, in illustration of my meaning, three fine specimens of calcification of the pulp, which had been forwarded to me by Dr. G. W. Matteson, of Michigan. The calcification in his tooth had apparently excited a certain amount of irritation in the remaining portion of the pulp, and this had been extended to the membranes covering the root and lining the alveolus. An application of aconite and iodine was made to the gum, which afforded decided relief to the patient. This application was repeated two or three successive mornings, with the same result. At the expiration of this time the patient, being engaged on an important case in court, was compelled to discontinue his visits to my office. Something like two or three weeks passed over without hearing from him, when I recived the following note:

"DEAR DOCTOR.—With your permission, I will stop at the office to-morrow, at half past eight. Is it possible that the solidification or ossification of that central pulp which you explained to me could have been accompanied by expansion, and thus have broken the tooth in two in its length? I think the fissure is there. Truly yours, G."

At the time appointed the gentleman made his appearance, and, on examination, a longitudinal fissure dividing the two cusps was readily perceived. The pain in the tooth during his absence had returned with increased intensity. All of a sudden it ceased, end then he discovered the fissure dividing the tooth into two halves.

After completing the examination, I remarked that my previous diagnosis had not been correct, that his difficulty had evidently been due to a devitalization of the pulp, from some cause or other, and that the dead pulp had undergone decomposition, and gases had been generated in the pulp cavity, which had exerted sufficient force to split the tooth.

I had heard and read of such cases before, in some of which the writers stated that the cleavage or bursting of the tooth had been attended with a loud explosive sound, seeming to the patient like the discharge of a pistol. This was the first case of the kind, however, which had ever come under my own observation. I advised the immediate removal of the tooth; and, as the patient did not desire to to suffer unnecessary pain, we called upon Dr. T.R. Thomas, who administered the nitrous oxide, and it was extracted while he was completely under the influence. After the removal, the fracture was found to extend longitudinally through the centre of the tooth to the point where the buccal and palatine roots bifurcated. The greater portion of the palatine root has been absorbed, while the bulbous portion of the pulp cavity was occupied by the remains of the putrescent pulp.

The case was very interesting and instructive to me, as I had been rather skeptical with regard to the description of cases of a similar character that I had met with heretofore. The evidence presented in this instance was so unmistakable and convincing, that I could arrive at no other conclusion than that the cleavage was due to the force exerted by the gases which had been formed in the pulp cavity.—*Dental Cosmos.*

---

